# Soil microbiomes in lawns reveal land-use legacy impacts on urban landscapes

**DOI:** 10.1007/s00442-023-05389-8

**Published:** 2023-06-08

**Authors:** Grant L. Thompson, Natalie Bray, Peter M. Groffman, Jenny Kao-Kniffin

**Affiliations:** 1 School of Integrative Plant Science, Cornell University, Ithaca, NY 14853 USA; 2 Advanced Science Research Center at the Graduate Center, Environmental Sciences Initiative, City University of New York, New York, NY 10031 USA; 3 Cary Institute of Ecosystem Studies, Millbrook, NY 12545 USA

**Keywords:** Biodiversity, LTER, Land-use legacy, Lawn, Soil microbiome, Urban ecological homogenization, Urban grassland

## Abstract

Land-use change is highly dynamic globally and there is great uncertainty about the effects of land-use legacies on contemporary environmental performance. We used a chronosequence of urban grasslands (lawns) that were converted from agricultural and forested lands from 10 to over 130 years prior to determine if land-use legacy influences components of soil biodiversity and composition over time. We used historical aerial imagery to identify sites in Baltimore County, MD (USA) with agricultural versus forest land-use history. Soil samples were taken from these sites as well as from existing well-studied agricultural and forest sites used as historical references by the National Science Foundation Long-Term Ecological Research Baltimore Ecosystem Study program. We found that the microbiomes in lawns of agricultural origin were similar to those in agricultural reference sites, which suggests that the ecological parameters on lawns and reference agricultural systems are similar in how they influence soil microbial community dynamics. In contrast, lawns that were previously forest showed distinct shifts in soil bacterial composition upon recent conversion but reverted back in composition similar to forest soils as the lawns aged over decades. Soil fungal communities shifted after forested land was converted to lawns, but unlike bacterial communities, did not revert in composition over time. Our results show that components of bacterial biodiversity and composition are resistant to change in previously forested lawns despite urbanization processes. Therefore land-use legacy, depending on the prior use, is an important factor to consider when examining urban ecological homogenization.

**Supplementary Information:**

The online version contains supplementary material available at 10.1007/s00442-023-05389-8.

## Introduction

Land-use change is a dominant component of global environmental change. While there has been great effort to understand the ecological and environmental implications of different land-use and land-cover types, less attention has been paid to effects of legacies of past land-use on the current conditions (Ziter et al. 2017). There is concern that these legacies are an important unexplored driver of the dynamics and environmental performance in many areas (Bürgi et al. 2017).

A dominant component of global land-use change is urbanization and expansion of landscape features that include urban grasslands, comprised of parcels of lawns (Robbins and Birkenholtz 2003; Pouyat et al. 2009; Wang et al. 2017; Ignatieva and Hedblom 2018). Lawns are an ecosystem of grasses that cover 2% of the terrestrial land in the U.S.—an area three times larger than any irrigated crop (Milesi et al. 2005). Though managed as fragmented parcels by individual landowners, urban grasslands are interconnected with limited physical barriers to the movement of biota both above and below ground, and thus function as intact ecosystems (Groffman et al. 2009; Raciti et al. 2011a; Durán et al. 2013; Polsky et al. 2014; Thompson and Kao-Kniffin 2017; Trammell et al. 2017). Lawns are often characterized by perennial cover and longer periods of photosynthesis and water uptake than forests or agricultural ecosystems (Pickett et al. 2008), high plant productivity (Falk 1980), and high soil microbial biomass and activity (Shi et al. 2012) which affect nutrient cycling and soil organic matter (Qian et al. 2010; Qian and Follett 2012). Soil carbon (C) content in urban soils has been shown to equal or surpass agricultural or native shortgrass ecosystems by comparison in both arid and mesic climates (Kaye et al. 2005; Golubiewski 2006; Raciti et al. 2011a, b), and turfgrass landscapes can have similar C stocks across ecoregions, including variations in climate, parent material, and topography (Pouyat et al. 2009). While urban grasslands have unique ecological features, they exist as a result of transformation and urbanization of a previous landscape, which could be influenced by legacy effects of land-use and of human management (Pickett et al. 2008; Pouyat et al. 2009).

Land-use change associated with urban development alters not only aboveground features, including vegetation, built structures, and impervious surfaces, but also soil physiochemical conditions such as pH, nutrient content, bulk density, and other factors, which affect biodiversity and soil biology (Pouyat et al. 2010, Pickett et al. 2011, von der Lippe et al. 2020). In urban grasslands, the use of fertilizers, supplemental irrigation, and disturbance by mowing generally select for soil microbiomes with a greater abundance of copiotrophic microorganisms that prefer higher resource environments (Thompson and Kao-Kniffin 2019; Sapkota et al. 2021). Studies have shown increasing bacterial abundance over time in residential lawns, but lawn management practice intensities and turfgrass species composition have not been linked to differences in microbial abundance (Acosta-Martinez et al. 1999; Shi et al. 2006; Yao et al. 2006; Allan-Perkins et al. 2019). Rather, the cumulative effects of irrigation, fertilization, and pesticide use on soil organic matter (SOM) and combined with soil texture (specifically silt) likely drive altered soil microbiome structure (Sapkota et al. 2021). It is consistent with other research showing that lawn management practices, including N fertilization and incorporation of leaf litter (Acosta-Martinez et al. 1999), are secondary determinants of soil microbiomes (Shi et al. 2006; Yao et al. 2006; Allan-Perkins et al. 2019; Sapkota et al. 2021). The well-studied nature of agricultural ecosystems has resulted in a proposed hierarchy of factors that influence the soil microbiome: soil type > time > specific farming operation > management system > spatial variation (Bossio et al. 1998). The study of urban ecosystems is complicated by many factors, inhibiting the development of a similar hierarchical framework for urban landscapes (Sapkota et al. 2021).

While urban ecosystems are located across very disparate climatic regions, the landscape features and management practices that typify an “urban” landscape may have resulted in the spread of ecologically similar ecosystems. The structural and functional resemblance of geographically distinct cities to one another, more so than to adjacent native ecosystems, refers to urban ecological homogenization (Groffman et al. 2014). While urban soils are often highly modified (IUSS Working Group WRB 2015), the soils in urban ecosystems often originate from the site. It is unclear how the process of homogenization is affected by remnant soil conditions that assert a legacy of previous use (Byrne et al. 2008; Pouyat et al. 2015; Ziter and Turner 2018). There is particular interest in the homogenizing effects on the phylogenetic structure of bacteria and fungi and their potential to perform ecosystem services, including nutrient cycling, pollution mitigation, and carbon sequestration in soils (Jangid et al. 2011).

In this study, we examined the biotic diversity of the soil ecosystem under land-use change from forest and agriculture to urbanized ecosystems in residential parcels of different age (spanning decades) and land-use history (forest versus agriculture), as well as reference sites of forests and agricultural lands. We aimed to examine if land-use history alters soil microbiome structure and soil physiochemical properties after recent conversion and through a span of several decades to determine if ecological homogenization occurs regardless of previous land-use. We hypothesized that conversion of forests and agricultural lands to residential lawns will lead to a disruptive shift in soil microbial composition that will remain distinctly different from the previous land-use but will be similar to other lawns regardless of previous land-use. We tested this hypothesis by focusing on specific taxonomic groups that are highly sensitive to the time-since-conversion into lawns. The underlying concept is that lawn soil microbiomes, regardless of previous land-use history, will become more similar over time, which is consistent with ideas about ecological homogenization of urban ecosystems. Lawn management activities, including establishing turfgrass species, mowing, fertility and water management, and recreational traffic, are expected to result in the reduction of soil legacy effects and the homogenization of soil microbiomes toward a common community.

## Methods

### Site selection and soil sampling

We determined land-use legacy impacts on the soil ecosystem by sampling from residential sites that were either previously forested or agricultural and compared them with present-day forest and agriculture reference sites. A total of 24 residential sites in Baltimore County, MD, USA were selected based on land-use history (agriculture or forest) and age (old: > 70 years; middle: 40–70 years; young: 10–25 years). There were four replicate lawns for each land-use and age combination. Four agricultural reference soils were derived from cropped land at the McDonogh School (Owings Mills, MD, USA) and four forest reference soils originated from Oregon Ridge Park (Baltimore County, MD, USA) (Appendix S1: Figure S1). Both sites are utilized as references for the other studies by the Baltimore Ecosystem Study (BES), as part of the National Science Foundation’s Long-term Ecological Research program (LTER) (Groffman et al. 2009). To identify the 24 residential properties used in this study, multiple data sources were compiled, and cross referenced to locate candidate sites prior to homeowner interviews and on-site assessments to determine the suitability of candidate properties for this research. Nine hundred residential lawns were identified from a 2011 telephone survey about lawn maintenance practices conducted by researchers affiliated with the BES LTER (Vemuri et al. 2011; Polsky et al. 2014). Combining land-use/land-cover classification data from the Maryland Department of Planning (State of Maryland 2015b), along with additional land-use/land-cover records (Wehling 2001) and ArcGIS (ESRI, Redlands, CA, USA) to assess individual parcel land-use history and real property records, maintained by the Maryland Department of Assessment and Taxation (State of Maryland 2015a) to determine the year of construction of the primary residential structure on each property, we created three age categories of candidate residential lawns: old, medium, and young for homes built in 1950 or prior, in the 1970s, and from 1990 to 2004, respectively. Homeowners were contacted via postal mail and in person via door-to-door canvassing. Before research sites were finalized, an on-site suitability assessment for lawn condition, tree shade, topography and drainage, and an interview with homeowners were conducted (data not reported). We attempted to standardize the selection of each property to be similar for these characteristics and to disperse sites geographically across the study region. This research builds on decades of work in the BES LTER that have characterized landscapes along an urban-to-rural gradient and studied residential property management practices by the homeowners in the region. Several extensive studies of randomly sampled properties in the Baltimore region found that most lawn soil profiles show no evidence of extensive use of fill material or soil profile disruption (Raciti et al. 2011a; Martinez et al. 2014). Additional research associated with the BES LTER has found that fertilizer and pesticide use in lawns is quite variable in time and space, but that the vast majority of lawns have received some fertilizer and pesticide over the past 25 years (Raciti et al. 2011b; Polsky et al. 2014; Locke et al. 2019). This study relies on prior data and findings from BES LTER research and methodologies used nationally by the LTER network to inform this study design, sampling scheme, and site selection.

At each residential site, two soil cores (3.2 cm dia × 100 cm depth) at least 4 m apart were collected using a slide hammer soil corer after removing grass and thatch layers (AMS, Inc., American Falls, ID, USA). Cores were divided into 0–30 cm and 30–100 cm increments, subsequent analyses reported here focus on the 0–30 cm depth increment. Further separation of the 0–30 cm core was not performed due to processing limitations of subsequent analyses. At the reference sites, two cores were taken from four plots at least 10 m apart at Oregon Ridge and McDonogh School. All cores were encased in individual plastic sleeves, capped and kept on ice in coolers, and returned to Cornell University (Ithaca, NY, USA) for subsequent analysis. Soil cores were removed from plastic sleeves, photographed, and examined for evidence of soil profile disruption or abrupt transitions that would indicate soil fill over native site soils (Raciti et al 2011a, b). The two replicate field cores from each site were combined in the lab and passed through a 2 mm sieve. Subsamples were frozen (− 20 °C until analysis) for microbiome biomarker sequencing and the remaining soil was divided for physiochemical analyses. Soil samples were frozen to stabilize the microbial community under a common set of conditions across all samples (Thompson and Kao-Kniffin 2016; Chou et al. 2018; Howard et al. 2020).

### Soil bacterial and fungal communities

DNA was extracted from 0.25 to 0.3 g of soil using the MoBio PowerSoil DNA Isolation Kit (MoBio Laboratories Inc. Carlsbad, CA, USA) per the manufacturer’s directions; amplification of the 16S rRNA gene (bacteria) and internal transcribed spacer (ITS) region (fungal) using universal primers and adapters for two-step Nextera library preparation for Illumina MiSeq sequencing (Howard et al. 2017). Amplification was performed using a Bio-Rad C1000 Thermo Cycler (Bio-Rad, Hercules, CA, USA). For the 16S rRNA gene, we used the primers 341F (5′-CCTACGGGNGGCWGCAG-3′) and 805R (5′-GACTACHVGGGTATCTAATCC-3′) (Herlemann et al. 2011), including index attachment overhangs (Bell et al. 2016; Howard et al. 2017). PCR reactions occurred in 20 μL volumes with 8 μL of 5 PRIME HotMasterMix (5 PRIME Inc., Gaithersburg, MD, USA), 1 μL of 10 μM concentrations of each primer, and 1 μL DNA template. Reactions were first performed with no dilution of the DNA template, then diluted to 1:5 and 1:10 with filter and steam sterilized, nuclease-free water until amplification was achieved. The 16S PCR was run as follows: 94 °C for 2 min; 25 cycles of 94 °C for 20 s, 55 °C for 20 s, and 72 °C for 30 s with a final elongation at 72 °C for 5 min. For ITS amplification, we used the primers ITS1F (5′-CTTGGTCATTTAGAGGAAGTAA-3′) and 58A2R (5′-CTGCGTTCTTCATCGAT-3′) (Gardes and Bruns 1993; Martin and Rygiewicz 2005) with index adapters as described above. ITS reactions occurred in 20 μL volumes with 8 μL of 5 Prime HotMasterMix, 1 μL of each primer in 10 μM concentrations, and 0.5 μL of DMSO. PCR conditions for ITS were 94 °C for 3 min; 35 cycles of 94 °C for 20 s, 45 °C for 30 s, and 72 °C for 45 s with a final elongation at 72 °C for 5 min. Multiple attempts at ITS PCR, including dilutions of 1:1, 1:5, and 1:10, and pooled triplicate extractions of the PowerSoil kit were unsuccessful in amplifying one replicate of the young lawns with agricultural history, and so, this sample was omitted from further cleaning and submission for sequencing.

PCR amplicons were cleaned using a MagBio HighPrep PCR magnetic bead kit (MagBio Genomics, Gathersburg, MD, USA) in a clear 96-well plate per the manufacturer’s directions. Using a 96-well plate, two unique barcode indices were added to the overhangs of each sample by combining 5 μL of amplicon, 2.5 μL of each forward and reverse primer containing the index barcode, 2.5 μL of polymerase free H_2_O, and 12.2 μL of Q5 High Fidelity 2X Master Mix (New England Biolabs, Inc., Ipswich, MA, USA). PCR cycling conditions were: 98 °C for 1 min; 8 cycles of 98 °C for 15 s, 55 °C for 30 s, and 72 °C for 20 s; with a final elongation at 72 °C for 3 min. The SequalPrep Normalization Kit (Thermo Fisher Scientific, Waltham, MA, USA) was used following the manufacturer’s instructions to normalize the DNA retained from each barcoded sample. We combined 5 μL of each normalized sample to separate 16S and ITS amplicon pools, which were concentrated and run on a 1.2% agarose gel for 5 min at 100 V and then 45 min at 60 V. Bands of the expected sizes were excised and the gel was removed from the sample using the PureLink Quick Gel Extraction Kit to give a final pooled volume of 50 μL for both 16S and ITS samples.

Pooled samples were run at the Cornell Genomics Facility (Ithaca, NY, USA) on the Illumina MiSeq platform with a 500-cycle MiSeq Reagent Kit v.2 for the ITS pool, and a 600-cycle MiSeq Reagent Kit v.3 for the 16S rRNA pool. A total of 505,301 16S rRNA gene and 648,940 ITS reads, representing 12,550 bacterial and 3125 fungal OTUs, respectively, were obtained following a paired-end merging, primer trimming, and singleton removal and sequence processing (Howard et al. 2017). MiSeq data have been deposited in the NCBI Sequence Read Archive and are available under the BioProject number SRP149939.

### Soil physiochemical properties

Approximately 20 g of fresh soil per soil core was dried at 100 °C for 72 h and reweighed to determine soil moisture content for subsequent calculations, including soil bulk density. Soil pH was measured using 10 g in a 1:2 solution with DI H_2_O which was shaken, then let stand for 30 min, and then read with a calibrated pH probe (Robertson et al. 1999). Due to the need to sample fresh soil for microbial analyses [community sequencing and potentially mineralizable nitrogen (PMN); PMN data not reported in this paper], the cores were sieved and homogenized and removed subsamples’ soil fresh weights were recorded. Materials passing and not passing the sieve were weighted fresh, dried, and reweighted. The dry weight of the microbial analyses’ fresh soil samples were calculated by the soil moisture content of the remaining materials. All subsamples of each core actual and calculated dry weights were divided by the core volume to determine soil bulk density. Subsamples were oven-dried at 72 °C for 24 h and ground into a fine powder using a mortar and pestle with liquid nitrogen (Cook et al. 2017) and analyzed at the Cornell Stable Isotope Laboratory (Ithaca, NY, USA) for total carbon and nitrogen analysis via an NC2500 elemental analyzer (Carlo Erba, Italy). Soil C and N stocks were calculated using total C and N, soil bulk density, core volumes, and the fraction of material greater than 2 mm (Raciti et al. 2011a, b). Soil texture was determined by the standard hydrometer sodium hexametaphosphate dispersal method described by Robertson et al. (1999) including no-soil calibration tests to account for solution density.

### Statistical analyses

Statistical analyses were performed in R (v. 3.6.2) (R Core Team 2017). Microbiome data were analyzed using the *phyloseq* (McMurdie and Holmes 2013) package after first being rarefied to yield an equal number of sequences in all samples. One replicate agricultural history, young lawn was removed from bacterial microbiome community analysis due to an inadequate number of Illumina sequences (< 1000 reads for the sample vs. 505,301 16S rRNA gene reads for the entire run). This is the same replicate that we were unable to successfully extract and amplify fungal DNA for ITS sequencing. This replicate was therefore removed from both bacterial and fungal community analysis, and soil physiochemical properties for this sample were not used for microbiome community analysis. Microbiome sequences were analyzed separately for 16S rRNA gene (bacteria) and ITS (fungi) using Analysis of Variance (ANOVA). Age of the lawn (young, medium, old, and reference) and land-use (previously agricultural or forested) were used for comparisons of means. Bray–Curtis dissimilarity matrices using the *vegan* package (Oksanen et al. 2018) were calculated for the bacterial and fungal sequences and permutational multivariate analysis of variance (PERMANOVA) using the *adonis* function in the *vegan* package were used to test the effect of land-use (forest or agricultural history) and age (young, medium, old, and reference) on bacterial and fungal community composition for all samples. Then, PERMANOVA was used to analyze the effect of age within sites of agricultural history and forest history separately. Non-metric multidimensional scaling (NMDS) on Bray–Curtis matrices was performed using the function *metaMDS* in the *vegan* package to visualize the differences in the microbial communities (stress values < 0.1). Soil physiochemical property effects on the microbial communities were calculated using the function *envfit* in the *vegan* package and visualized using vectors plotting the correlations to the NMDS ordinations. Significance values for the vectors were generated with 999 permutations.

Shannon diversity indices using the *diversity* function and relative abundance using the *phyloseq* function for bacterial and fungal communities were calculated in *vegan* package and differences were assessed using linear models tested with ANOVA followed by Tukey’s HSD post hoc to assess the effects of land-use history (forest or agricultural) and age (young, medium, old, and reference) together, or the effect of age within separate agricultural history and forest history analyses. The Shannon index was used, because the value increases as the number of species increases, and the evenness of species distribution among samples becomes more even, which has been shown to be more reliable than the Simpson index for microbiome research (Pylro et al. 2014).

The *lsmeans* package was used to fit linear models to test the effects of land-use (lawn, forest, and agriculture) on soil physiochemical properties (pH, bulk density, soil texture, C and N stocks, and C:N). Where needed, data were transformed to meet normality assumptions. Model significance was tested by analysis of variance (ANOVA). Post hoc pairwise comparisons were then made using Tukey’s HSD test using the *cld* function in *lsmeans*. Data with *p* < 0.05 were considered significant.

## Results

### Bacterial 16S rRNA gene sequences

#### Bacterial composition

**Fig. 1:**
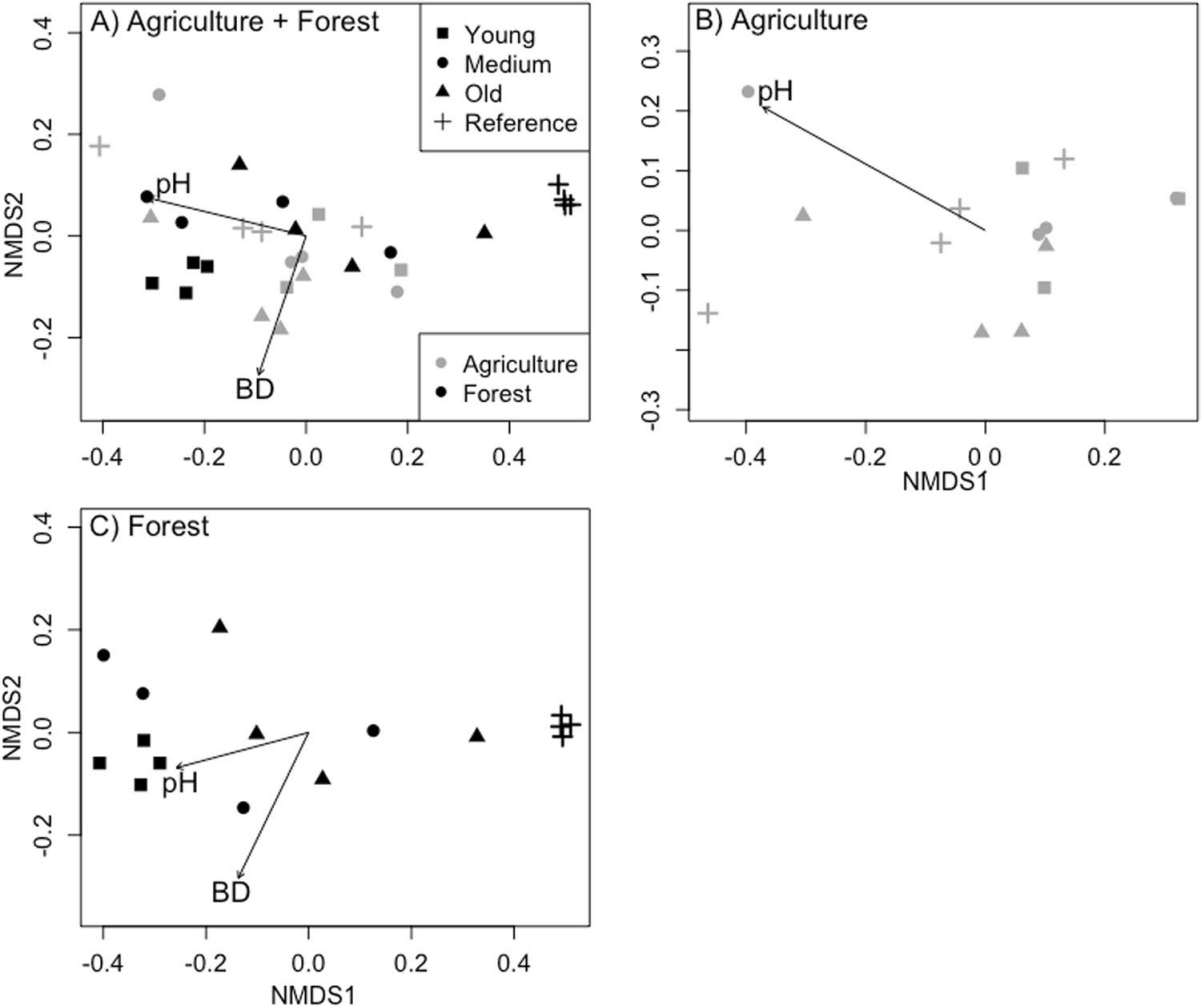
Soil bacterial composition in lawns that were previously agricultural or forested. Non-metric multidimensional scaling (NMDS) was used to show variation in bacterial composition using 16S rRNA gene sequences for soils spanning young, medium, and old residential sites. The contrasting land-uses are combined in the top ordination (**A**) and are shown separately in **B** as lawns converted from agriculture and **C** lawns converted from forests. The ordination includes soils from reference sites that are presently agricultural or forested. Vectors represent soil physiochemical properties, pH, and bulk density (BD) that were significantly correlated with the ordinations. PERMANOVA at *p* < 0.05 was used to determine distinct microbial communities across residential age and reference sites

Bacterial microbiomes were compared across all sites, which showed that while land-use history and age factors were significant, separation of the dataset by land-use history improved analysis of the age factor (PERMANOVA: land-use history: *F*_1,29_ = 6.24, *R*^2^ = 0.16, *p* = 0.001; age: *F*_3,29_, = 2.31, *R*^2^ = 0.18, *p* = 0.001) (Fig. 1A). The dataset was then divided to evaluate shifts in microbial community composition with age within each land-use history. For lawn soils that were previously agriculture, soil pH influenced the clustering of bacterial communities from sites that served as agricultural references in this study (pH vector: *p* = 0.001; NMDS: stress value = 0.089, non-metric *R*^2^ = 0.99), while residential age or the time-since-conversion to lawns from prior agricultural sites was not a significant predictor of bacterial composition (PERMANOVA: *F*_3,11_ = 0.99, *R*^2^ = 0.21, *p* = 0.44) (Fig. 1B). However, distinct shifts in bacterial communities across lawn age were visible for previously forested lawn soils (PERMANOVA: *F*_3,12_ = 5.45, *R*^2^ = 0.58, *p* = 0.001). There was a shift in bacterial composition from reference forests to young lawns along NMDS1 (*x*-axis; NMDS: stress value = 0.055, non-metric *R*^2^ = 0.99) (Fig. 1C). Bacterial community shifts are shown as clusters by age of residences—young lawns are distinctly separated from reference forests, while older lawns are more similar to reference forests. Both pH and bulk density were correlated with the ordination of the bacterial community (pH: *p* = 0.017; BD: *p* = 0.004).

#### Bacterial diversity

Shannon diversity of the soil bacterial communities differed across land-uses and lawn age (two-factor ANOVA: *F*_7,23_ = 8.89, *p* = 2.69 × 10^–5^) (Table 1). Shannon diversity was not significantly different across the lawns of various ages and the reference site within the agricultural land-use history group of sites (one-factor ANOVA: *F*_3,11_ = 0.22, *p* = 0.88). However, within the forest land-use history sites, Shannon diversity of the forest reference site was significantly lower (mean: 5.60, sd: 0.10) compared to all other samples (one-factor ANOVA: *F*_3,12_ = 23.45,* p* = 2.63 × 10^–5^).

**Table 1:** Shannon diversity of bacteria and fungi

Land-use	Lawn age	*n*	Shannon diversity bacteria		Shannon diversity fungi	
			Land-use × age (two-factor ANOVA)	Age within land-use (one-factor ANOVA)	Land-use × age (two-factor ANOVA)	Age within land-use (one-factor ANOVA)
Agriculture	Young lawn	3^a^	6.43 ± 0.16*a*	6.43 ± 0.16*A*	2.83 ± 0.34*c*	2.83 ± 0.34*A*
	Medium lawn	4	6.56 ± 0.27*a*	6.56 ± 0.27*A*	3.68 ± 0.51*ab*	3.68 ± 0.51*A*
	Old lawn	4	6.53 ± 0.20*a*	6.53 ± 0.20*A*	3.66 ± 0.32*ab*	3.66 ± 0.32*A*
	Reference	4	6.45 ± 0.33*a*	6.45 ± 0.33*A*	3.31 ± 0.24*abc*	3.31 ± 0.24*A*
Forest	Young lawn	4	6.41 ± 0.18*a*	6.41 ± 0.18A*	4.01 ± 0.25*a*	4.01 ± 0.25A*
	Medium lawn	4	6.71 ± 0.14*a*	6.71 ± 0.14A*	3.60 ± 0.21*abc*	3.60 ± 0.21AB*
	Old lawn	4	6.54 ± 0.31*a*	6.54 ± 0.31A*	3.56 ± 0.12*abc*	3.56 ± 0.12B*
	Reference	4	5.60 ± 0.10*b*	5.60 ± 0.10B*	3.00 ± 0.27*bc*	3.00 ± 0.27C*

Values are mean ± SD of untransformed values. The two-factor ANOVA included all measurements across both land-use histories (agriculture and forest) and one-factor ANOVA analyses were done separately for lawns with agriculture history and lawns with forest history with their respective reference sites. Measurements not connected by the same letter and the same case indicate means were significantly different according to a post hoc Tukey’s HSD test (lowercase letters for the two-factor ANOVA, uppercase letters for the one-factor ANOVA, and within uppercase letters, asterisks for forest land-use)

^a^One replicate young lawn with agricultural history did not amplify for sequencing, and thus, this replicate was omitted from both bacterial and fungal diversity analysis

#### Bacterial relative abundances

**Fig. 2:**
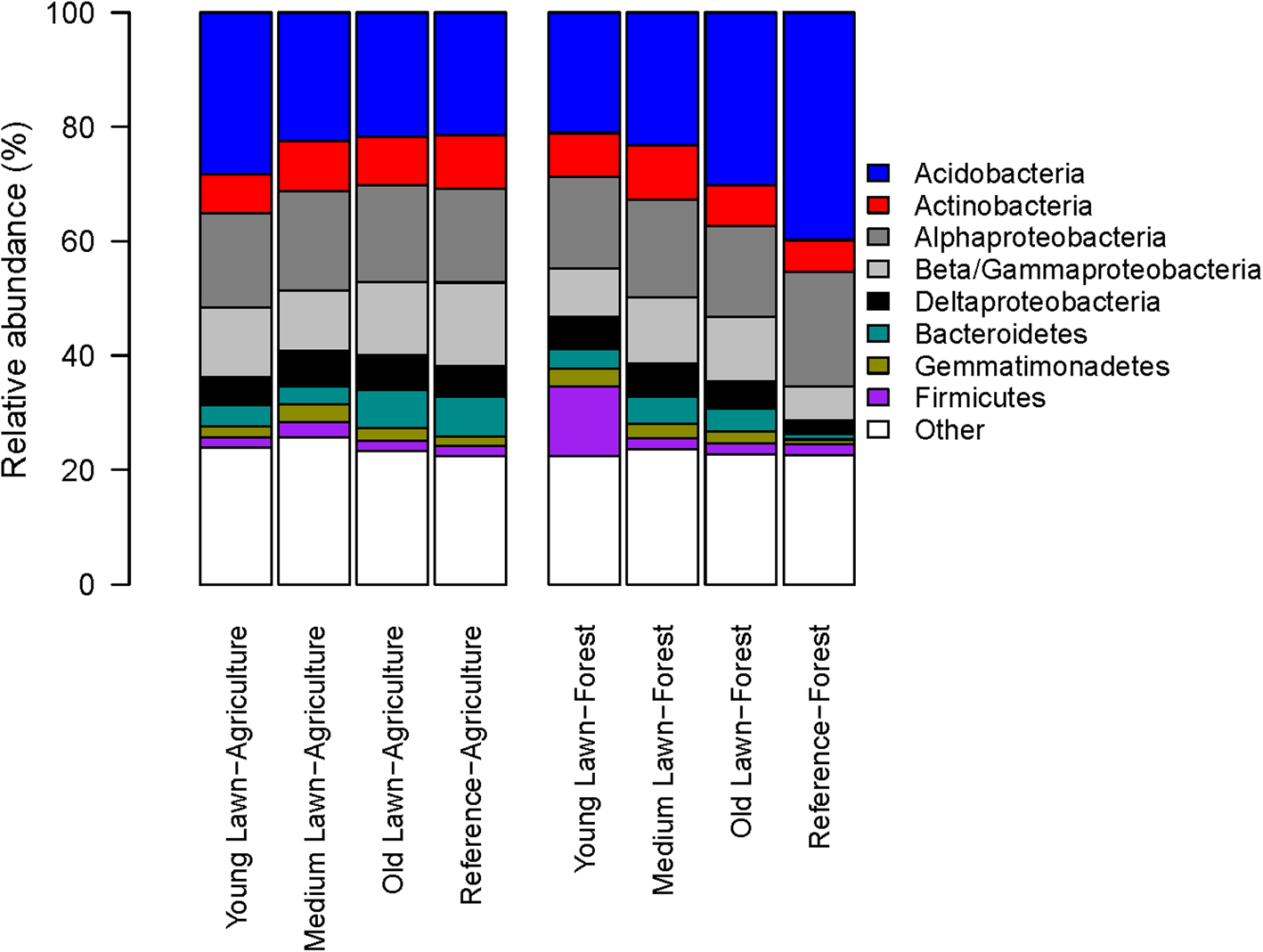
Relative abundances of dominant bacterial taxonomic groups (phylum and class) based on 16S rRNA gene sequences. The stacked bars on the left indicate soils from lawns that were previously agricultural and the bars on the right are derived from lawns previously forested. Reference sites that are presently agricultural or forested are included for comparison with residential soils

Reference forest soils were distinguished by a higher relative abundance of taxa in the phylum *Acidobacteria* (mean: 39.81, sd: 2.19; ANOVA: *F*_7,23_ = 8.06, *p* = 5.73 × 10^–5^) and a lower relative abundance of taxa in the phylum *Gemmatimonadetes* (mean: 0.77, sd: 0.19; *F*_7,23_ = 13.08, *p* = 1.1 × 10^–6^) (Fig. 2). Furthermore, previously forested young lawns had a higher relative abundance of the taxa in the phylum *Firmicutes* (mean: 12.18, sd: 4.05; ANOVA: *F*_7,23_ = 18.20, *p* = 5.48 × 10^–8^). The *Actinobacteria* (*F*_7,23_ = 1.64, *p* = 0.174), *Alphaproteobacteria* (*F*_7,23_ = 0.85, *p* = 0.56), *Beta/Gammaproteobacteria* (*F*_7,23_ = 2.26, *p* = 0.066), and *Deltaproteobacteria* (*F*_7,23_ = 2.09, *p* = 0.087) did not vary across land-uses and lawn age.

When regressed against soil pH, the *Acidobacteria* increased in relative abundance (Appendix S1: Figure S1) with greater soil acidity in both previously agricultural (*F*_1,13_ = 8.73,* p* = 0.011) and forest soils (*F*_1,14_ = 7.30*, p* = 0.017), while the *Alphaproteobacteria* increased in only the previously forested sites (*F*_1,14_ = 4.74, *p* = 0.047) (Supplemental Table S1). In contrast, as soil pH increased, there was a corresponding rise in relative abundances of *Deltaproteobacteria* in both land-use histories (previously agricultural: *F*_1,13_ = 16.93, *p* = 0.0012; previously forested: *F*_1,14_ = 11.09, *p* = 0.005) and only in previously forested sites for *Beta/Gammabacteria* (*F*_1,14_ = 5.61, *p* = 0.033), *Bacteroidetes* (*F*_1,14_ = 6.75, *p* = 0.021), and *Gemmatimonadetes* (*F*_1,14_ = 9.77, *p* = 0.0075).

### Fungal ITS region

#### Fungal composition

**Fig. 3:**
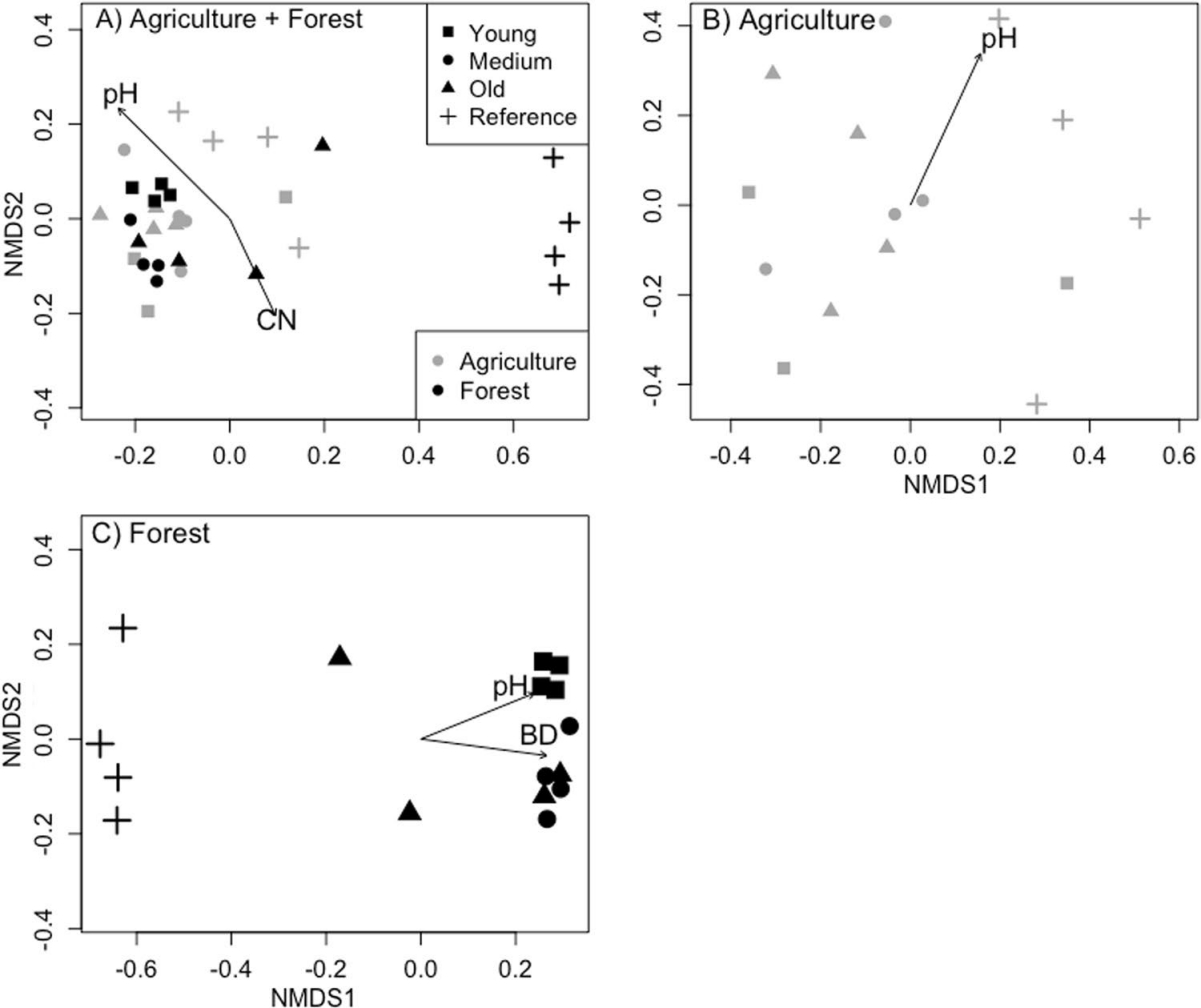
Soil fungal composition in lawns that were previously agricultural or forested. Non-metric multidimensional scaling (NMDS) was used to show variation in fungal composition using ITS gene sequences for soils spanning young, medium, and old residential sites. The contrasting land-uses are combined in the top ordination (**A**) and are shown separately in **B** as lawns converted from agriculture and **C** lawns converted from forests. The ordination includes soils from reference sites that are presently agricultural or forested. Vectors represent soil physiochemical properties, pH, and bulk density (BD) that were significantly correlated with the ordinations. PERMANOVA at *p* < 0.05 was used to determine distinct microbial communities across residential age and reference sites

Soil fungal composition, assessed via ITS region sequencing, followed many similar patterns to trends of the soil bacterial communities. With the combined land-use analysis, soil fungal community composition was significantly different among land-uses (residential lawns, reference forest sites, and reference agriculture sites; PERMANOVA: *F*_2,28_ = 2.53, *R*^2^ = 0.15, *p* = 0.001). Fungal communities of the reference forests were distinct from all other land-uses, and young lawns that were previously forested clustered together and diverged from reference sites along NMDS1 of the ordination (NMDS: stress value = 0.12, non-metric *R*^2^ = 0.99) (Fig. 3A). Soil physiochemical properties that were significantly correlated to the fungal community ordination included pH (*p* = 0.001) and C:N (*p* = 0.031).

For lawn soils in previously agricultural lands, residential site age was not a significant predictor of fungal composition (PERMANOVA: *F*_3,11_ = 1.09, *R*^2^ = 0.23, *p* = 0.17) (Fig. 3B). Soil pH influenced the direction of fungal composition in the ordination (pH vector: *p* = 0.025; NMDS: stress value = 0.17, non-metric *R*^2^ = 0.98). Fungi in forested sites and previously forested lawns varied by age (PERMANOVA: *F*_3,15_ = 2.81, *R*^2^ = 0.41, *p* = 0.001) with a gradient along NMDS1, where previously forested young and medium lawns differed from forested reference sites and the older lawns were less distinct from forested reference sites (NMDS: stress value = 0.046, non-metric *R*^2^ = 0.99) (Fig. 3C). Both pH and bulk density were significantly correlated with the fungal community ordination (pH: *p* = 0.022; BD: *p* = 0.020).

#### Fungal diversity

Shannon diversity of the soil fungi differed across land-uses and lawn age (ANOVA: *F*_7,23_ = 5.95, *p* = 0.00049) (Table 1). Within the agriculture land-use history group of sites, Shannon diversity differed among the lawn ages and the agricultural reference site (one-factor ANOVA: *F*_3,11_ = 3.87, *p* = 0.041). Within the forest land-use history group, Shannon diversity differed to a stronger degree among the lawn ages and the reference forest than among the agricultural-history sites (one-factor ANOVA: *F*_3,12_ = 13.85, *p* = 0.00033). The youngest residential sites showed different levels of fungal diversity, with greater diversity in the previously forested lawns (mean: 4.01, sd: 0.25) and the lowest in the previously agriculture lawns (mean: 2.83, sd: 0.34). Soil fungal composition became more diverse with increasing age in former agricultural lands.

#### Fungal relative abundances

**Fig. 4:**
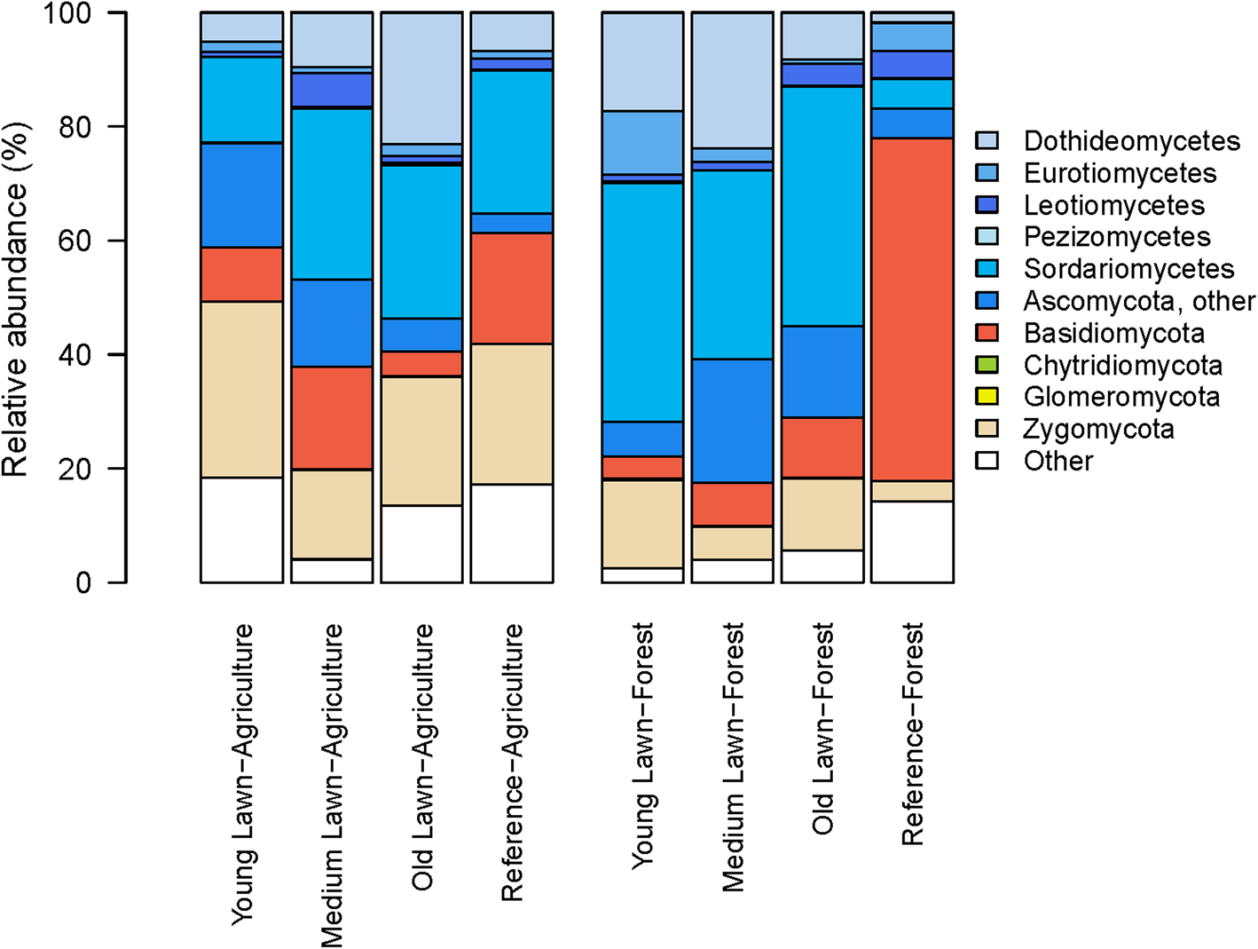
Relative abundances of dominant fungal taxonomic groups (phylum and class) based on ITS gene sequences. The stacked bars on the left indicate soils from lawns that were previously agricultural and the bars on the right are derived from lawns previously forested. Reference sites that are presently agricultural or forested are included for comparison with residential soils

There were variations in the relative abundances of the phylum *Basidiomycota* and classes of the phylum *Ascomycota,* including *Sordariomycetes*, *Eurotiomycetes*, *Dothideomycetes*, and unclassified *Ascomycota* (“Ascomycota, other”) across land-use and lawn age (ANOVA, Basidio: *F*_7,23_ = 7.64, *p* = 8.55 × 10^–5^, Sordar: *F*_7,23_ = 3.27, *p* = 0.015, Euro: *F*_7,23_ = 3.29, *p* = 0.014, Doth: *F*_7,23_ = 3.37, *p* = 0.013, other Asco: *F*_7,23_ = 4.19, *p* = 0.0041) (Fig. 4). The reference forest sites had a higher relative abundance in *Basiodiomycota* (mean: 60.14, sd: 18.40) and lower relative abundance of other *Ascomycota* (mean: 22.11, sd: 9.22) compared to previously forested lawns, but did not differ from agriculture reference sites and previously agriculture lawns. Previously forested young and old lawns had higher relative abundance of *Sordariomycetes* (previously forested young lawns: mean: 41.81, sd: 6.69 and previous forest old lawns: mean: 42.11, sd: 20.36) compared to the forest reference site (mean: 5.21, sd: 2.84). The younger lawns that were previously forested had higher relative abundance of *Eurotiomycetes* (mean: 11.24, sd: 9.01) compared to all the agricultural-history lawns and agriculture reference sites and the previously forested old lawns. The forest reference sites had lower relative abundance of *Dothideomycetes* (mean: 8.18, sd: 6.60) compared to the former agriculture old lawns (mean: 23.04, sd: 17.82) and medium-aged formerly forested lawns (mean: 23.87, sd: 13.04). *Leotiomycetes* (*F*_7,23_ = 1.63, *p* = 0.18), *Pezizomycetes* (*F*_7,23_ = 1.17, *p* = 0.36), *Chytridiomycota* (*F*_7,23_ = 1.43, *p* = 0.24), *Glomeromycota* (*F*_7,23_ = 2.04 *p* = 0.093), and *Zygomycota* (*F*_7,23_ = 1.02, *p* = 0.45), did not vary with land-use and lawn age.

When regressed against soil pH, the relative abundances of unclassified *Ascomycota* increased in association with greater soil acidity (Appendix S1: Figure S3) in previously agricultural sites (*F*_1,13_ = 10.17, *p* = 0.0071) and *Basidiomycota* in previously forested sites (*F*_1,14_ = 9.88, *p* = 0.0072) (Appendix S1: Table S2). Conversely, there was a strong positive association between *Sordariomycetes* and less acidic soils in previously forested sites (*F*_1,14_ = 28.68, *p* = 0.00010).

### Soil properties: pH, bulk density, texture, and carbon and nitrogen stocks

Soil pH, bulk density (BD), texture (% sand, % silt and % clay), C and N stocks, and C:N were compared across reference sites (agriculture and forest) and residential lawns (Appendix S1: Table S3). Soil pH was significantly lower in the forest reference sites compared to all other sites (mean: 4.44, sd: 0.11; *F*_3,28_ = 6.789, *p* = 0.001). Soil percent sand (*F*_3,28_ = 2.116, *p* = 0.120), silt (*F*_3,28_ = 1.154, *p* = 0.345) and clay (*F*_3,28_ = 1.612, *p* = 0.209) were not significantly different among land-uses. Soil bulk density ranged from 1.00 to 1.45 g cm^–3^ and was not significantly different between land-uses (*F*_3,28_ = 1.364, *p* = 0.274). There were no significant differences in soil C stock (*F*_3,28_ = 1.873, *p* = 0.157), N stock (*F*_3,28_ = 1.402, *p* = 0.263), or C:N (*F*_3,26_ = 0.429, *p* = 0.733) among different land-uses.

## Discussion

We examined if land-use legacy influences the trajectory of biotic homogenization in soil ecosystems upon conversion from forest or agriculture to urban grasslands. In this study, temporal trends in microbiome community dynamics were uncovered only when assessing previous land-use. In previously forested sites, bacterial and fungal communities were patterned across time-since-conversion to residential lawns. For example, with increasing age of the residences, the bacterial communities of previously forested lawns were reverting to a forest soil composition over decades of time, but the soil fungi did not follow a similar pattern to bacteria. Additionally, the agricultural lands converted to lawns showed no shifts in the soil microbial community over time.

The convergence of microbiomes across lawns and agricultural sites, with the exception of the bacteria found in the old previously forested lawns, suggests that the homogenizing effects of urbanization (Groffman et al. 2014) are similar to the effects of other human-managed ecosystems (e.g., agriculture) in altering soil microbiomes and edaphic conditions (Ziter and Turner 2018). The homogenizing effect across human-managed ecosystems may be due to the similarities in landscape features and management practices (e.g., presence of non-woody vegetation, fertilization, and disturbance) in residential lawns and agricultural sites. A global analysis of microbiomes across urban green spaces revealed more homogeneous microbial communities across cities than those found across in reference, adjacent natural ecosystems (Delgado-Baquerizo et al. 2021). The study supports the concept that biotic homogenization is captured in the microbial sequence record, but the factors leading to homogenization at the global scale are unclear. In our study of the Baltimore ecosystem watershed, we found that the previously agricultural sites undergo a significant transformation into residential lawns, but the disturbance is not detectable via soil bacterial and fungal sequence data or in the outcome of many soil physiochemical properties. Knowing previous land-use history can partly explain the variability in responses, but without more extensive data on management interventions, discerning predictable outcomes will remain difficult (Ziter and Turner 2018). The intersection of land-use legacy and ecological resilience to disturbance is evident in the microbiome fingerprint of the contrasting ecosystems. The *Acidobacteria*, in particular, may be at the core in resisting the ecological homogenization of lawns by reestablishing populations over time (Fig. 2). The Acidobacteria are comprised of metabolically diverse taxa, with specific subdivisions associated with acidic or low pH soils (Kielak et al. 2016). The bacteria are common in forests soils and are found in the rhizospheres of grass species (Pan et al. 2014). While the overall microbial community may appear similar across sites, slight variations in microbial taxa could have impacts on specific ecosystem functions.

The unique microbiome of the forest reference sites was likely influenced by the acidic soil conditions, evidenced by the lower bacterial and fungal diversity levels and changes in relative abundances of *Acidobacteria*, *Proteobacteria*, and *Bacteroidetes* in the bacterial communities and *Basidiomycota* and *Sordariomycetes* in the fungal communities. It is well known that soil pH is one of the primary drivers of microbial community composition, and diversity across ecosystems and within urban ecosystems (Fierer and Jackson 2006; Lauber et al. 2009; Rousk et al. 2010; McGuire et al. 2013; Ramirez et al. 2014; Schmidt et al. 2017). Soil pH can influence bacterial communities through direct effects on nutrient availability or ion toxicity and indirect effects of maintaining cellular activity and metabolism in acidic environments (Zhalnina et al. 2015). The *Acidobacteria*, *Actinobacteria*, *Bacteroidetes*, and *Firmicutes* have been shown to be responsive to soil pH (Lauber et al. 2009; Wessén et al. 2010). Although, in this study, pH was not correlated with *Actinobacteria* or *Firmicutes,* suggesting that there are likely other factors affecting their population dynamics in our sites (Appendix S1: Figure S2 and Table S1).

The *Proteobacteria* and *Bacteroidetes* were also less abundant in the reference forest plots. These bacterial phyla include many copiotrophic species that grow in environments with abundant nutrients and resources. The taxonomic composition of the forest soil microbiomes indicates an environment favoring more oligotrophic (Hartman and McCarthy 2008; Cederlund et al. 2014; Zhang et al. 2017) microbiota than the other sites examined in this study. Deeper analysis of the nutrient profiles in soil, including forms of soil organic matter and plant residues, may elucidate the differences in microbial communities across land-uses (Cederlund et al. 2014). Nonetheless, our results highlight those forests within an urban–suburban area are in fact distinct from other land-uses at the microbiome level. The unknown factor is why the soil bacterial community in urban residences becomes more similar to those found in forests, while soil fungi do not exhibit the same trend over time in these residential sites.

A massive disturbance, like deforestation and replacement of vegetation from trees to grass, could alter soil fungi structure permanently, even if remnant trees remain or if new ones are established in older urban landscapes. In our analysis, *Basidiomycota*, as expected, dominated reference forest fungal communities. Members of this phylum perform important functions that include the breakdown of lignin (Taylor and Sinsabaugh 2015; Anthony et al. 2017), which is a significant component of organic matter in the forested sites. The *Sordariomycetes* and *Dothideomycetes,* classes of fungal phylum *Ascomycota*, are associated with the decomposition of leaf litter (Šnajdr et al. 2011; Taylor and Sinsabaugh 2015), and thus, their greater abundance in previously forested lawns compared to previously agricultural lawns could result from litter inputs or relative tree cover in the surroundings. This concept is also consistent with the increasing abundance of these classes of fungi on more mature lawns of formerly agricultural sites, which may have more woody landscape plantings established following conversion to residential development which likely increases leaf-litter inputs with time as those trees mature. Leaf litter inputs for sites of both land-use histories would be expected to increase with time as residential landscape trees mature, though sites with a prior forest history may receive additional leaf-litter input from adjacent landscapes. However, unlike forest ecosystems where coarse woody debris would remain in place, supporting the decomposer food web and microbiome, woody debris is removed from most residential landscapes and lawns, so the differential outcomes for microbial wood-decomposers and leaf-decomposers is consistent with our findings across time and former land-use history.

Bulk density was particularly important for microbial communities in the forested sites, as indicated by its significant correlation with NMDS microbial community ordinations. The difference in bulk density in the forest sites (1.14 g × cm^−1^) and formerly forested lawns (1.23 g × cm^−1^) indicate that even a small increase in bulk density, as a result of land-use change, can affect bacterial community composition. Bulk density is a direct measure of particle packing and accounts for air/water exchange, pore spaces, and void spaces influencing microbial and root establishment and persistence (Li et al. 2002; Canbolat et al. 2006). Our results indicate that the soil microbiome may be sensitive to bulk density in residential lawns. While many studies have highlighted that ecosystem edaphic properties, such as pH and bulk density, are predictive of continental scale patterns of soil microbiomes (Fierer and Jackson 2006; Lauber et al. 2009), functional changes in soil microbiomes that may occur as a result of changes in vegetation, litter and soil organic matter at the smaller scale may not be captured by these commonly reported metrics. As soil microbiome studies across urban and other ecosystems continue to provide more information on the links between microbial communities and soil physiochemical properties, land-use history, and age, since conversion could explain some of the inconsistencies in relating these two variable types.

There was a high proportion of the bacterial phylum *Firmicutes* in the lawn soils that were most recently converted from forest, which comprised approximately 12% of the relative abundance of the entire soil bacterial community. In contrast, *Firmicutes* was only 1.7–2.9% of the total soil bacterial relative abundances in all other land-uses and ages. The properties with high *Firmicutes* abundance were widely distributed across the study region [Fig. 1, young (Y) and forest land-use history], and therefore, we do not suspect that a proximity effect is driving this finding. Other studies report *Firmicutes* relative abundance at 2–5% (Lauber et al. 2009) across many ecosystem types. In a recent census of bacterial taxa in urban grasslands along the eastern mid-Atlantic region of the United States, including Beltsville, MD (Crouch et al. 2017), *Firmicutes* abundances ranged from 1.9 to 4.8%. Evidence from > 40 year old continually grazed rye-grass pastures (Lauber et al. 2008) and the long-running Rothamsted Park Grass experiment (PGE) (Zhalnina et al. 2015), report *Firmicutes* relative abundances in the range of 13–15%. Generally, *Firmicutes* are reported as copiotrophic (Zhalnina et al. 2015), although they have been shown to be positively associated with ammonium sulfate fertilizer, though this is not universal for all genera within the phylum (Zhalnina et al. 2015). *Firmicutes* tend to be negatively correlated with pH (Lauber et al. 2008; Wessén et al. 2010), which aligns with the reported stimulatory effect of ammonium sulfate fertilizer on *Firmicutes* relative abundance, since ammonium sulfate acidifies soils. We found no significant correlation between *Firmicutes* relative abundance and soil pH, %C, %N, or C:N (Appendix S1: Figure S2, Table S1, and Table S4). Prior to the PGE, the site was a permanent grazed pasture until 1875 and it was suggested by researchers that the abundance of gut-associated microbiota (*Firmicutes*) may be a centuries old land-legacy effect at the PGE. Although, the authors suggested that modern era small mammals, birds, and soil fauna may also be sources of animal-associated microbiota (Zhalnina et al. 2015). Given the high *Firmicutes* abundance occurring in younger lawns with a forest history, animal inputs cannot be ruled out or accounted for if the soil were external to the sites and brought to the residences during construction. The presence of animal-associated microbiota in grass-dominated landscapes requires further research, particularly if there may be human health implications.

While bacterial diversity did not differ across the agriculture-history lawns and agriculture reference sites, fungal diversity was lowest in the young lawns with agricultural history. While fungal sequencing has many known limitations and biases against phylogenetic groups (e.g., *Glomeromycota* (Stockinger et al. 2010), this lower diversity in younger lawns indicates a disturbance effect as a result of the transformation from an agricultural site. Fungi are in fact sensitive to belowground disturbances (Treseder et al. 2004; Jansa et al. 2006; van der Heyde et al. 2017). Therefore, during the transformation from agriculture to a grass lawn, it is important to consider the ecosystem services provided by fungi, such as nutrient cycling and soil aggregation that may be impacted during the younger transition phase.

Though residential lawn care practices in the Baltimore, MD (USA) region are variable in space and time (Raciti et al. 2011a; Polsky et al. 2014; Locke et al. 2019), the effects of irrigation, fertilization, mowing, turfgrass species composition, and soil disturbance can affect soil biogeochemistry and soil microbiome composition and function (Thompson and Kao-Kniffin 2019). While lawn management practices can affect the soil microbiome, such effects are secondary to soil properties and time (Acosta-Martinez et al. 1999; Allan-Perkins et al. 2019; Sapkota et al. 2021), where soil properties set the bounds for which management can influence the microbiome unless management practices are directly attempting alter soil properties. Compared to unmanaged ecosystems, lawn landscapes—even those that received minimal management inputs (i.e., municipal lawns and right of ways)—are at least periodically mown, and many residential lawns receive periodic irrigation or fertilization (Locke et al. 2019; Thompson and Kao-Kniffin 2019). The increased availability of resources in residential lawn ecosystems from fertilization and irrigation tends to broadly select for copiotrophic microorganisms (Leff et al. 2015; Zhalnina et al. 2015; Thompson and Kao-Kniffin 2019). Soil disturbance after lawn establishment has been shown to have minimal effects on soil microbiomes adapted to typical edaphic and lawn management conditions (Yao et al. 2006; Bartlett et al. 2007; Crouch et al. 2017). Moreover, soil and soil microbiome import associated with sod installation at the U.S. National Mall lawn renovation have been shown not to appreciably disturb existent soil microbiomes and that long-term management practices are what selects for the observed soil microbiome (Crouch et al. 2017).

The conversion of land to urban use implies not only significant disturbance during the development and construction process of urbanization, but also in the continued management of human-dominated landscapes (Groffman et al. 2017; Locke et al. 2019; Thompson and Kao-Kniffin 2019). We acknowledge, as have others (Ziter and Turner 2018; Sapkota et al. 2021), that conducting urban ecology research is difficult in part because of the lack of detailed site history and human management activities that may influence many factors—including the soil microbiome that we considered in our study. However, the strength of our experimental design was that we used historic aerial imagery to create a clear contrast between sites with agricultural versus forest land-use history. The fact that there are observable legacy effects of this contrast, despite variation in soil and plant management in lawns, is perhaps our most significant result. This study is associated with and builds on decades of work within the Baltimore Ecosystem Study Long-Term Ecological Research (BES LTER) project. Findings from extensive prior BES LTER research help to address some of the unknowns and possible assumptions about the comparability of randomly selected residential lawn sites used in this study, specifically that the majority of prior lawn soil profile research shows little evidence of fill or soil profile disruption (Raciti et al. 2011b; Martinez et al. 2014) and while variable in time and space the vast majority of lawns have received some fertilizer and pesticide over the past 25 years (Raciti et al. 2011a; Polsky et al. 2014; Locke et al. 2019). Prior research has characterized an extensive ecological homogenization of lawn ecosystems across the U.S. (Groffman et al. 2017) and the fact that in our study we can still find the legacy effects of past land-use is quite striking and important for understanding multiple functions of these ecosystems (e.g., carbon storage, greenhouse gas fluxes, and water quality).

## Conclusion

Lawns are dominant features of urban and suburban landscapes; however, they are more recent features resulting from the transformation of a different land-use type. The lawn soil microbiome is understudied, yet this underlies the environmental performance of urban grasslands. Our results show that soil microbiomes are influenced by the previous land-use. Characteristics related to historic land-use, such as vegetation type and management interventions, affect the structure and diversity of present soil bacterial and fungal communities and potentially their ecological performance. Our study also supports the urban ecological homogenization hypothesis in that lawn establishment and management practices result in largely identical soil microbiomes regardless of previous land-use. We hypothesize that this change likely occurs at the time of construction or within a few years after, though our results, based on our study design, show homogenization with a decade or 2 after conversion. However, the decades of time-since-conversion showed contrasting developments in microbiome structure in once forested landscapes. With increasing age of the residences, soil bacterial communities became more similar to reference forests. While bulk soil did not become more acidic over time in these previously forested residences, soil pH influenced the relative abundances of specific key taxa.

Overall, the results indicate that the legacy effects of the previous land-use may be residual in soil over decades of time to impact long-term trajectories of biodiversity and urban ecological homogenization. Our findings show evidence of shifting soil microbiomes that differ based on time and land-use history in response to contemporary lawn management. This finding may not have direct implications for homeowners presently, but it does suggest that changes in the soil microbiome linked to landscape management may result in shifts in the function of these landscapes. There is great interest in the ecosystem services and disservices related to air and water quality associated with residential land-use. The processes that influence these services are mediated by microorganisms and there is a great need to understand how land-use history, management, and other factors influence microbial communities and processes.

## Supplementary Information

Below is the link to the electronic supplementary material.

Supplementary file1 (PDF 327 KB)

## Data Availability

Bacterial 16S rRNA gene and fungal 16S ITS MiSeq data have been deposited in the NCBI Sequence Read Archive and are available under the BioProject number SRP149939.
