# Supplementary material for: Soil microbiomes in lawns reveal land-use legacy impacts on urban landscapes
Source: Oecologia. 2023 Jun 8;202(2):337–51. doi: 10.1007/s00442-023-05389-8 (PMC10307846; doi:10.1007/s00442-023-05389-8)
Supplement: Supplementary file 1 — Supplementary file1 (PDF 327 KB) [file 442_2023_5389_MOESM1_ESM.pdf]

Supplementary materials

Title: Soil microbiomes in lawns reveal land-use legacy impacts on urban landscapes

Authors: Grant L. Thompson [1], Natalie Bray[1], Peter M. Groffman[2], and Jenny Kao-Kniffin[1*]

[1]School of Integrative Plant Science, Cornell University, Ithaca, NY, 14853

[2]City University of New York, Advanced Science Research Center at the Graduate Center, Environmental Sciences Initiative, New York, NY, 10031, and Cary Institute of Ecosystem Studies, Millbrook, NY 12545

Journal: *Oecologia*

Table S1: Results of linear models assessing the relationships between pH and relative abundance of bacterial community phyla for agriculture history lawns and reference sites and forest history lawns and reference sites. Asterisks indicate significant p- values (* <0.05; ** <0.01).

**Relative abundance (%)**	**Agriculture history lawns and reference sites pH**		**Forest history lawns and reference sites pH**	
	**F value**	**p-value**	**F value**	**p-value**
Acidobacteria	$F_{1,13} = 8.73$	0.011*	$F_{1,14} = 7.30$	0.017*
Actinobacteria	$F_{1,13} = 0.12$	0.74	$F_{1,14} = 1.52$	0.24
Alphaproteobacteria	$F_{1,13} = 3.79$	0.074	$F_{1,14} = 4.74$	0.047*
Deltaproteobacteria	$F_{1,13} = 16.93$	0.0012*	$F_{1,14} = 11.09$	0.0050**
Beta/Gammabacteria	$F_{1,13} = 2.35$	0.15	$F_{1,14} = 5.61$	0.033*
Bacteroidetes	$F_{1,13} = 1.26$	0.28	$F_{1,14} = 6.75$	0.021*
Gemmatimonadetes	$F_{1,13} = 0.043$	0.84	$F_{1,14} = 9.77$	0.0075**
Firmicutes	$F_{1,13} = 0$	0.99	$F_{1,14} = 0.78$	0.39

Table S2: Results of linear models assessing the relationships between pH and relative abundance of fungal community phyla for agriculture history lawns and reference sites and forest history lawns and reference sites. Asterisks indicate significant p- values (* <0.05; ** <0.01, *** <0.001).

**Relative abundance (%)**	**Agriculture history lawns and reference sites pH**		**Forest history lawns and reference sites pH**	
	**F value**	**p-value**	**F value**	**p-value**
Dothideomycetes	$F_{1,13} = 2.87$	0.11	$F_{1,14} = 0.99$	0.34
Eurotiomycetes	$F_{1,13} = 0.26$	0.62	$F_{1,14} = 0.69$	0.42
Leotiomycetes	$F_{1,13} = 3.58$	0.081	$F_{1,14} = 2.33$	0.15
Pezizomycetes	$F_{1,13} = 1.72$	0.21	$F_{1,14} = 0.95$	0.35
Sordariomycetes	$F_{1,13} = 0.29$	0.60	$F_{1,14} = 28.68$	0.00010***
Ascomycota, other	$F_{1,13} = 10.17$	0.0071**	$F_{1,14} = 0.51$	0.49
Basidiomycota	$F_{1,13} = 2.66$	0.13	$F_{1,14} = 9.88$	0.0072**
Chytridiomycota	$F_{1,13} = 2.12$	0.17	$F_{1,14} = 0.51$	0.49
Glomeromycota	$F_{1,13} = 0.003$	0.96	$F_{1,14} = 2.38$	0.15
Zygomycota	$F_{1,13} = 0.53$	0.48	$F_{1,14} = 1.08$	0.32

Table S3: Soil properties to 1 m depth. Values are mean±SD of untransformed values. Measurements not connected by the same letter indicate means were significantly different according to a post-hoc Tukey's HSD test.

**Land-use and lawn age**	**n**	**pH**	**BD** (g * cm^{-3})	**Sand** (%)	**Silt** (%)	**Clay** (%)	**C stock** (kg C * m^{-2})	**N stock** (g N *m^{-2})	**Soil C:N** (ratio)
Young Lawn Agriculture History	4	5.86 ±0.51 *a*	1.28 ±0.11 *a*	38.02 ±13.38 *a*	35.46 ±10.23 *a*	26.52 ±3.31 *a*	4.58 ±1.03 *a*	441.00 ±101.05 *a*	8.70 ±1.08 *a*
Medium Lawn, Agriculture History	4	5.38 ±0.38 *ab*	1.06 ±0.08 *b*	42.12 ±18.47 *a*	30.96 ±16.25 *a*	26.93 ±14.69 *a*	3.70 ±1.12 *a*	357.75 ±96.62 *a*	10.68 ±6.72 *a*
Old Lawn, Agriculture History	4	5.75 ±0.24 *a*	1.15 ±0.06 *ab*	44.70 ±6.44 *a*	31.24 ±12.33 *a*	24.06 ±8.28 *a*	5.41 ±1.69 *a*	505.05 ±104.91 *a*	10.01 ±4.38 *a*
Agriculture Reference	4	6.02 ±0.59 *a*	1.21 ±0.09 *a*	58.60 ±7.90 *a*	24.29 ±3.90 *a*	17.10 ±4.27 *a*	6.45 ±3.08 *a*	463.80 ±48.66 *a*	11.76 ±1.92 *a*
Young Lawn, Forest History	4	5.69 ±1.01 *a*	1.27 ±0.09 *a*	53.88 ±18.32 *a*	23.63 ±9.93 *a*	22.50 ±11.28 *a*	3.97 ±0.87 *a*	337.58 ±71.55 *a*	11,72 ±1.84 *a*
Medium Lawn, Forest History	4	5.28 ±0.54 *a*	1.19 ±0.06 *ab*	37.05 ±9.58 *a*	37.66 ±6.38 *a*	25.29 ±4.20 *a*	4.59 ±2.16 *a*	347.63 ±113.34 *a*	14.04 ±5.58 *a*
Old Lawn, Forest History	4	6.16 ±0.16 *a*	1.22 ±0.02 *ab*	59.98 ±7.41 *a*	22.52 ±5.50 *a*	17.50 ±2.34 *a*	4.84 ±1.08 *a*	522.53 ±125.65 *a*	8.45 ±0.89 *a*
Forest Reference	4	4.44 ±0.11 *b*	1.14 ±0.05 *ab*	43.97 ±5.81 *a*	30.81 ±2.09 *a*	25.22 ±3.94 *a*	4.17 ±0.19 *a*	322.65 ±23.80 *a*	9.90 ±1.63 *a*

Table S4: Results of linear models assessing the relationships between soil physiochemical properties (% carbon, % nitrogen and C:N) and relative abundance of the bacterial phylum *Firmicutes* for agriculture history lawns and reference sites and forest history lawns and reference sites.

**Relative abundance (%)**	**Agriculture history lawns and reference sites**		**Forest history lawns and reference sites**	
	**F value**	**p-value**	**F value**	**p-value**
	**% carbon**		**% carbon**	
Firmicutes	$F_{1,13}$ = 0.067	0.80	$F_{1,14}$ = 0.96	0.35
	**% nitrogen**		**% nitrogen**	
	$F_{1,13}$ = 0.12	0.74	$F_{1,14}$ = 0.15	0.70
	**C:N**		**C:N**	
	$F_{1,13}$ = 1.66	1.42	$F_{1,14}$ = 0.78	0.25

Figure Legends

Figure S1: Figure S1: Locations of residential lawn sites (n = 24) and reference agricultural and forested sites in Baltimore County, Maryland, USA (39°24’ N 76°36’W). The sites are located near the City of Baltimore in the Gywnn Falls watershed and are part of the National Science Foundation Long-term Ecological Research (NSF LTER) focus on urban watershed dynamics of Baltimore.

Figure S2: Relationships between relative abundance of bacterial phyla and pH for lawns with agriculture history and agriculture reference sites (yellow) and for lawns with forest history and forest reference sites (green). Solid lines represent significant linear relationships between relative abundance of the indicated bacterial phylum and pH with p-values and R^2 values noted in corresponding color.

Figure S3: Relationships between relative abundance of fungal phyla and pH for lawns with agriculture history and agriculture reference sites (yellow) and for lawns with forest history and forest reference sites (green). Solid lines represent significant linear relationships between relative abundance of the indicated bacterial phylum and pH with p-values and R^2 values noted in corresponding color.

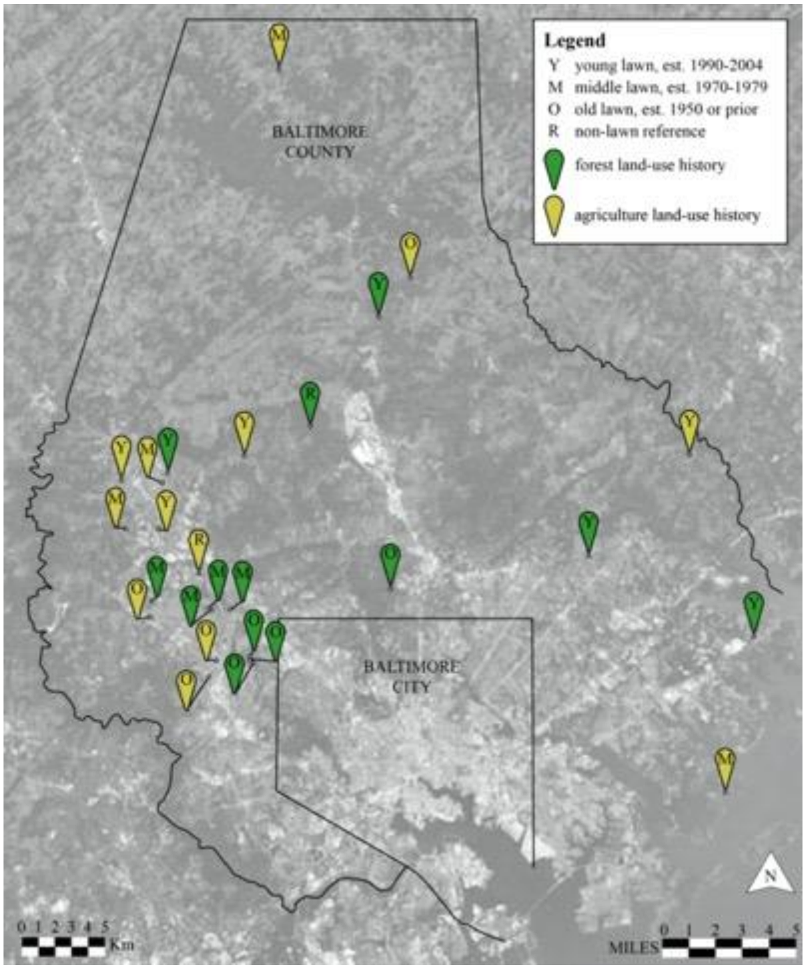

Figure S1: Locations of residential lawn sites (n = 24) and reference agricultural and forested sites in Baltimore County, Maryland, USA (39°24’ N 76°36’W). The sites are located near the City of Baltimore in the Gywnn Falls watershed and are part of the National Science Foundation Long-term Ecological Research (NSF LTER) focus on urban watershed dynamics of Baltimore.

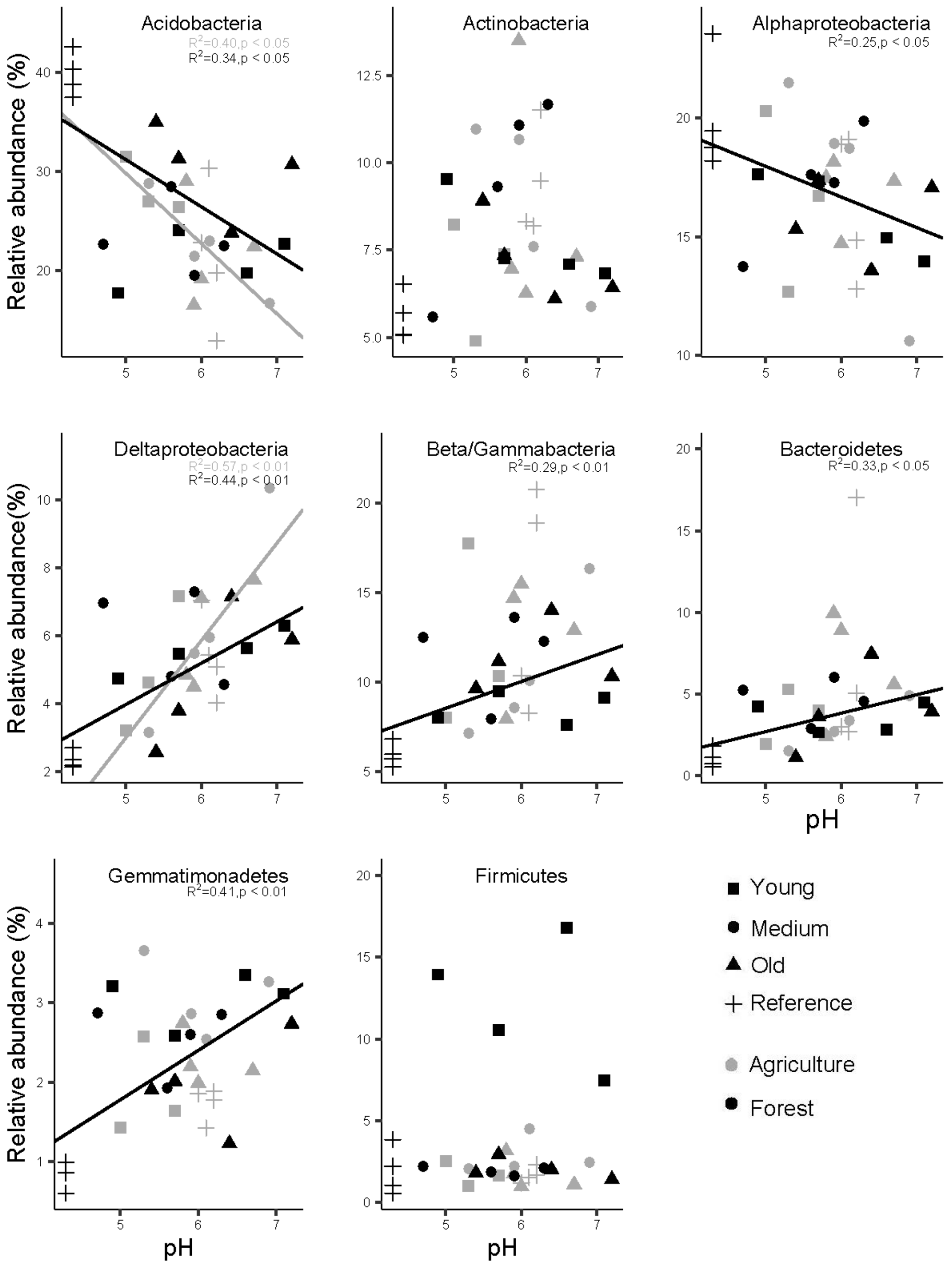

Figure S2: Relationships between relative abundance of bacterial phyla and pH for lawns with agriculture history and agriculture reference sites (yellow) and for lawns with forest history and forest reference sites (green). Solid lines represent significant linear relationships between relative abundance of the indicated bacterial phylum and pH with p-values and R^2 values noted in corresponding color.

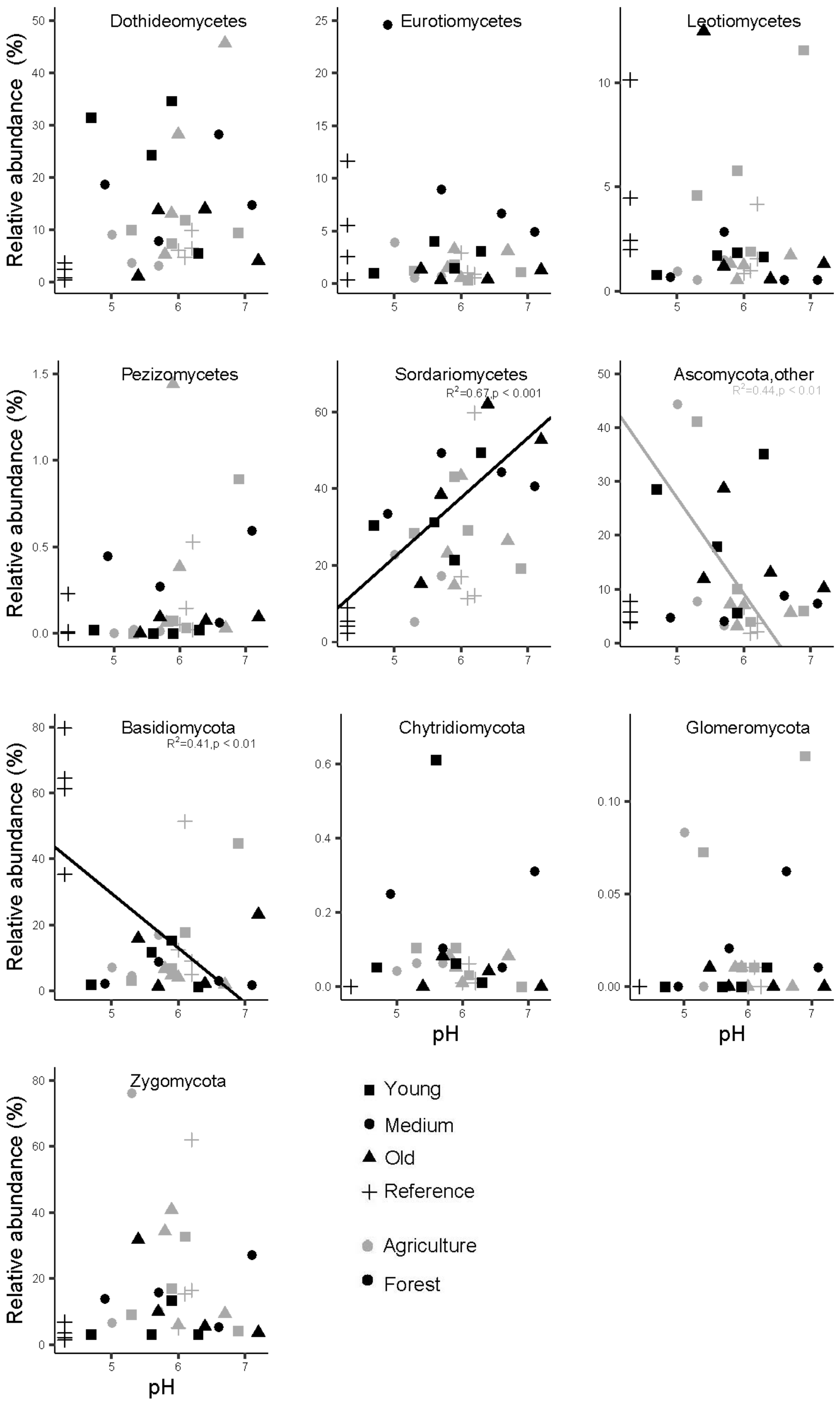

Figure S3: Relationships between relative abundance of fungal phyla and pH for lawns with agriculture history and agriculture reference sites (yellow) and for lawns with forest history and forest reference sites (green). Solid lines represent significant linear relationships between relative abundance of the indicated bacterial phylum and pH with p-values and R^2 values noted in corresponding color.
